# Recruitment to trials: insights from a meta-ethnography of qualitative studies

**DOI:** 10.1186/1745-6215-14-S1-O69

**Published:** 2013-11-29

**Authors:** Sharon McCann, Marion Campbell, Vikki Entwistle

**Affiliations:** 1 Health Services Research Unit, University of Aberdeen, Aberdeen, UK

## 

It is well known that recruitment to trials can be difficult. A number of in-depth qualitative studies have been published that have examined patient experiences of recruitment and participation in trials. To understand the collective insights from these studies, we undertook a meta-ethnography (a formal synthesis method for constructing interpretations cumulatively across qualitative studies) of qualitative studies published between 1996 and 2010, focusing on people's *own* accounts of their decisions to accept or decline trial participation. Our synthesis highlighted how key aspects of the context, recruitment approach and individual approached can interact to influence trial recruitment. The way potential participants were situated in terms of their health states and treatment junctures was particularly salient. Their perceptions of their situation at the time of being trial entry influenced their judgements about the implications of trial participation for both their own and the broader common good. These perceptions could mediate the influence of individuals communication and relationship with trial recruiters, of the nature of trial interventions and processes, their desire to help others, and of what their non/participation might say about them as persons. The synthesis and model of factors likely to affect recruitment will be presented.

